# A shape-optimized framework for kidney segmentation in ultrasound images using NLTV denoising and DRLSE

**DOI:** 10.1186/1475-925X-11-82

**Published:** 2012-10-30

**Authors:** Fan Yang, Wenjian Qin, Yaoqin Xie, Tiexiang Wen, Jia Gu

**Affiliations:** 1 Shenzhen Institutes of Advanced Technology, Chinese Academy of Sciences, Shenzhen, China; 2 Shenzhen Key Laboratory for Low-cost Healthcare, Shenzhen, China; 3 Graduate University of Chinese Academy of Sciences, Beijing, China

## Abstract

**Background:**

Computer-assisted surgical navigation aims to provide surgeons with anatomical target localization and critical structure observation, where medical image processing methods such as segmentation, registration and visualization play a critical role. Percutaneous renal intervention plays an important role in several minimally-invasive surgeries of kidney, such as Percutaneous Nephrolithotomy (PCNL) and Radio-Frequency Ablation (RFA) of kidney tumors, which refers to a surgical procedure where access to a target inside the kidney by a needle puncture of the skin. Thus, kidney segmentation is a key step in developing any ultrasound-based computer-aided diagnosis systems for percutaneous renal intervention.

**Methods:**

In this paper, we proposed a novel framework for kidney segmentation of ultrasound (US) images combined with nonlocal total variation (NLTV) image denoising, distance regularized level set evolution (DRLSE) and shape prior. Firstly, a denoised US image was obtained by NLTV image denoising. Secondly, DRLSE was applied in the kidney segmentation to get binary image. In this case, black and white region represented the kidney and the background respectively. The last stage is that the shape prior was applied to get a shape with the smooth boundary from the kidney shape space, which was used to optimize the segmentation result of the second step. The alignment model was used occasionally to enlarge the shape space in order to increase segmentation accuracy. Experimental results on both synthetic images and US data are given to demonstrate the effectiveness and accuracy of the proposed algorithm.

**Results:**

We applied our segmentation framework on synthetic and real US images to demonstrate the better segmentation results of our method. From the qualitative results, the experiment results show that the segmentation results are much closer to the manual segmentations. The sensitivity (SN), specificity (SP) and positive predictive value (PPV) of our segmentation result can reach 95%, 96% and 91% respectively; As well as we compared our results with the edge-based level set and level set with shape prior method by means of the same quantitative index, such as SN, SP, PPV, which have corresponding values of 97%, 88%, 78% and 81%, 91%, 80% respectively.

**Conclusions:**

We have found NLTV denosing method is a good initial process for the ultrasound segmentation. This initial process can make us use simple segmentation method to get satisfied results. Furthermore, we can get the final segmentation results with smooth boundary by using the shape prior after the segmentation process. Every step enjoy simple energy model and every step in this framework is needed to keep a good robust and convergence property.

## Background

US imaging is widely used in the area of diagnosis, image-guided interventions and therapy. It has the advantages of real-time capabilities and low cost compared with X-ray, computed tomography (CT) and magnetic resonance imaging (MRI). The accurate segmentation of organs or objects from US image plays a key role in its applications. However, compared with other medical imaging modalities, such as CT and MRI, US image is particularly difficult to do segmentation since the quality of the image is relatively low [1]. There are characteristic artifacts such as attenuation, speckle, shadows, and signal dropout which makes the segmentation task be complicated [2]. Moreover, kidney segmentation of ultrasound image is rarely studied in certain fields, such as [1] regarding the noise as a part of texture feature,which has to be extracted in every segmentation process due to diverse clinical scenes.

Recent years, many people use level set to conduct the segmentation. Since level set was proposed by Osher and Sethian, many models have been embedded in level set functions such as global intensity statistics [3], texture models [1], shape prior models [4] and Markov random field models [5] to improve the result. However, complicated computation in the level set evolution result in longer computing time. Furthermore, some methods have some drawbacks in themselves, such as texture models [1], shape prior models [4] have not taken into consideration of the problem when the shape prior training set of is not enough for the segmentation.

In this paper, we presented a novel framework for kidney segmentation in ultrasound (US) images to counter aforementioned limitations. This framework was combined with NLTV image denoising, DRLSE [6] and shape prior for initial process, segmentation process and post optimization process. In the initial process, in order to reduce the bad influence of noise, we used the effective NLTV image denoising to get an almost homogenous intensity gray scale image of kidney region. In the segmentation process, we used the DRLSE method to get the coarse segmentation results. DRLSE is a simple level set formulation to reduce the segmentation cost time. The main PDE equation contains two items. The first item detects object boundaries from image gradients. The second part is used to maintain the signed distance property | ∇ *Φ*| = 1. Thus, it needs not reinitialize the level set function (LSF).

However, the noise cannot be eliminated completely by use of the NLTV denoising method. Thus, the result of DRLSE method may also be influenced by the noise. Therefore, in the final post process, we used the shape prior to get a kidney shape space on the monochrome image produced by DRLSE to optimize the segmentations result of DRLSE.

Statistical modeling is one of the most used methods to model shape priors. Cootes et al. [7,8] proposed an active shape model (ASM) which relies on the statistics of an object’s shape gathered from a training set of manually land-marked instances of the object. They developed a parametric point distribution model for describing the segmenting curve. And the automatic placement of landmarks was presented in [9] and [10]. Reference [11] proposed a novel technique for solving the crucial correspondence problem of automatic placement of landmarks using non-rigid image registration. But this representation does not contain any explicit information about the point connectivity and landmarks are often obtained manually, which is a time-consuming, error-prone and subjective tendency.

Chen et al., [12], represented shapes using a collection of points. They applied clustering method instead of statistical method to get the shape prior model which is the average shape of given curves with similar shape, but different size, orientation and translation. However, the similarity of shapes in this method is measured by area information which makes it highly time-consuming. Rousson and Paragious [13] showed a method to recover a segmentation map in accordance with the shape prior model as well as a rigid registration between the map and the model, which is based on global-to-local registration [14] and prior region statistical properties. The advantage of this method is accounting for local degrees of variability and local shape variations, but it consists of N*N variables, and thus, is unstable. Using the Gabor filter bank to characterize the prostate boundaries in a multiscale and multiorientation pattern, Shen et al. [15] proposed a statistical shape model for the automatic prostate segmentation in transrectal US images.

In this paper, we adopted the proposed method in [4] and [16] to construct the kidney shape space. Leventon et al. [4] proposed a segmentation method including two steps: initial segmentation and its correction based on a shape prior model. The model is obtained through a principle component analysis (PCA) operation on a collection of signed distance maps of the training shapes. In this framework, the boundaries are represented as the zero level set of a 2-D scalar function [4]. This representation is intrinsic (independent of parameterizations) and is topologically flexible since different topologies of the curve are represented by the constant topology of scalar function. This characteristics is valuable for realistic image-guided diagnosis. In addition, when the sensitivity (SN), specificity (SP) and positive predictive value (PPV) of the segmentation results were all under 90%, we used the alignment model [16] to avoid the situation of lacking enough training shape sample for the segmentation. As a result, the shape space became richer and richer during the segmentation process compared with methods in [4,7,17,18] without applying the alignment model after the segmentation to enrich the shape space.

## Results

**Figure 1:**
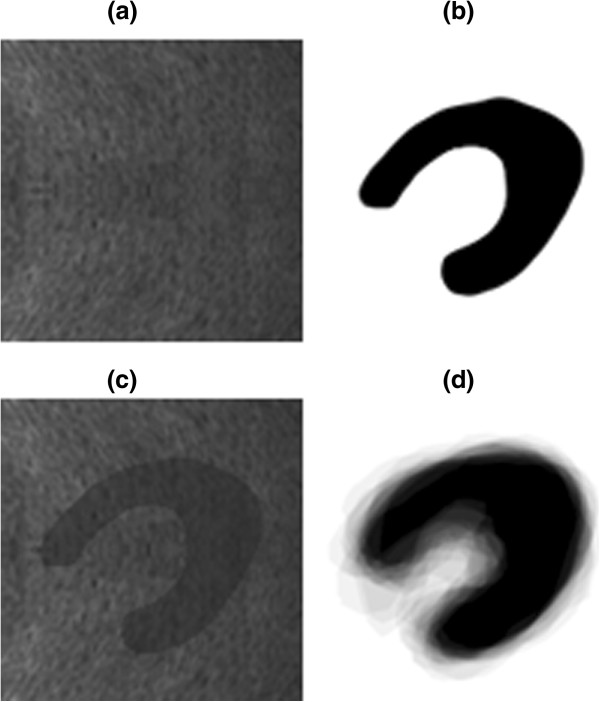
**Synthetic image and initial shape model. **(**a**) ultrasound noise image; (**b**) a shape come from the kidney shape space; (**c**) the mixture image of the noise image and shape image; (**d**) initial shape model.

**Figure 2:**
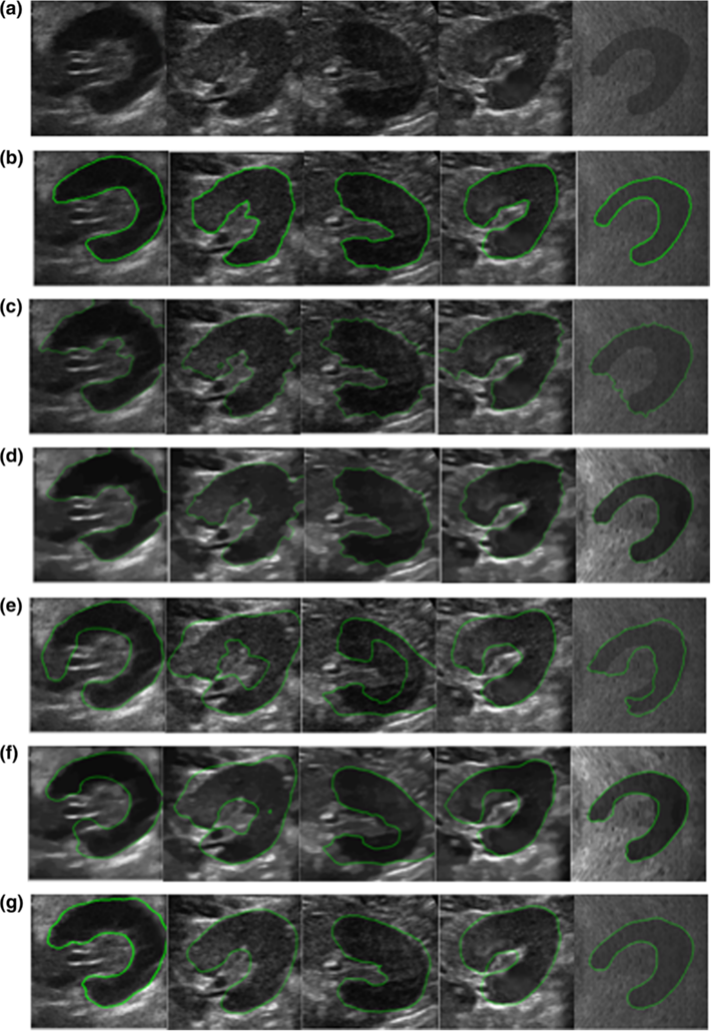
**Qualitative segmentation results of five methods for kidney in US image. **(**a**) original images; (**b**) manual segmentations(in green); (**c**) DRLSE method segmentations; (**d**) DRLSE segmentations on the NLTV denoised image; (**e**) Bression’s method segmentations; (**f**) Bression’s method segmentation on NLTV denoised image; (**g**) the results of our method.

**Figure 3:**
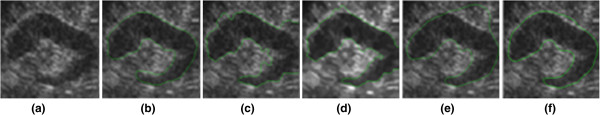
**Qualitative segmentation results of four methods for kidney in US image. **(**a**) original images; (**b**) manual segmentations (in green); (**c**) DRLSE method segmentations; (**d**) DRLSE segmentations on the NLTV denoised image; (**e**) the result of our method without using the alignment model after the segmentation; (**f**) the result of our method with using the alignment model after the segmentation.

For our experiments, several evaluations were performed on synthetic and real US images. The specific synthetic and real US images have 512×512 pixels. The real US images of left kidney were acquired by DC-7 ultrasound machine from Mindray with covex array transducers. So we need do extracted ROI of kidney before using our framework. The synthetic image (c) in Figure 1 is mixture of a shape image (b) in Figure 1, from our shape space and the noise image (a) in Figure 1. The real US images is shown in row (a) of Figure 2 and (a) of Figure 3. The training set of the shape space was also used in [1]. Our initial shape model was also used in [1], which is shown in image (d) in Figure 1. We used 11 eigenvectors to cover the 98.8% variation of the shape space. 

### Qualitative results

Our goal was to accurately and quickly delineate the boundaries of the kidney from the synthetic and US image in row (a) of Figure 2. We used four different segmentation algorithms to do comparison analysis. The segmentation results by manual method, DRLSE method, DRLSE method on NLTV denoised image, Bression’s method, Bression’s method on NLTV denoised image and our method were shown in rows (b), (c), (d), (g), (h), (e) in Figure 2 respectively. The manual delineations were got by the surgeon using ITK-SNAP software. From the results, we can see that NLTV denoising is essential in segmentation of US image, and DRLSE with shape prior is very effective. The segmentation results by our method were very close to the manual segmentation results.

The NLTV denoising is indispensable for ultrasound image processing. The initial process of images row (c) in the Figure 2 uses Gaussian low pass filter. Moreover, the initial process of images row (d) in Figure 2 uses the NLTV denoising. From the segmentation results, we can see that the NLTV dennoising method helps the DRLSE method get a better result. Because the NLTV denoising method deal with the every paths use the different weights, which makes the smooth path with homogeneity gray scale and the paths with large gradients to keep its boundary.

When using the level set with shape prior, it’s better to use the shape prior in the end of curve evolution. The initial process of the row (f) and (g) in Figure 2 use the NLTV denoising method. The row (f) use the common level set with shape prior method. The row (f) in Figure 2 uses the shape prior in the end of the curve evolution. We can see that using the shape prior in the end of curve evolution gives an accuracy result in the same initial process. Because use the shape prior in every level set iteration leads to the energy model getting convergence in the local minimum. Once we use the shape prior in the end of the level set evolution, both the level set process and the shape prior process gets its global minimum to make the level set with the shape prior get its global minimum.

In Figure 3, the experiment results show how we deal with the situation where the shapes in the shape space may be not enough. The segmentation results by manual method, DRLSE, DRLSE on NLTV denoised image, our method without using the AM after the segmentation and our method with using the AM after the segmentation were illustrated in rows (b), (c), (d), (e), (f). From Figure 3, it demonstrates that our method may not achieve the satisfied result occasionally, which is due to lack of sufficient kidney shapes from the shape space. Therefore the alignment model was used to absorb new shape to make the kidney shape space cover more shapes. Image (f) in Figure 3 is the segmentation result by our method with the initial shape prior, we can see that the segmentation results is not good. Because the kidney shape of image (a) in Figure 3 is not existing in the shape space. So we use the alignment model to absorb the new shape to get the new shape prior. Image (h) in Figure 3 shows the segmentation result with the new shape prior. After the alignment model had been applied, the accuracy of segmentation results were improved obviously.

It is worth mentioning that the total cost time is only around 10s(32-bit desktop PC:2.93 GHz Core 2 and 2GB RAM). The cost time of initial process, segmentation process and optimization process is 0.01s, 8s and 2s respectively, while it needs 5 min cost time of the level set with shape method in [17] and 40s cost time in [1].

### Quantitative results

For each image, the set of pixels lying within the manual delineations of kidney was denoted as *M*. The set of pixels lying within any boundary resulting from any method was denoted as *A*. We used SN, SP and positive PPV as the quantitative metrics of the segmentation results. SN, SP and PPV were defined as:

$\text{SN}=\frac{\left|M\cap A\right|}{\left|M\right|},\text{SP=}\frac{\left|W\right|-\left|M\cup A\right|}{\left|W\right|-\left|M\right|},\text{and PPV=}\frac{\left|M\cap A\right|}{\left|A\right|}$, where *W* denoted the whole image, | · | denoted the point set of the segmentation result. We computed SN, SP and PPV for each image as well as the average and standard deviation over the experiment data set. Higher value of these metrics means that the segmentation results is closer to the manual segmentations. The statistics result is shown in Table 1. The difference of SN, SP and PPV between using and not using the alignment model in the segmentation process is shown in Table 2. From the Table 1 and Table 2, we can see that the better result we get in the Qualitative result region, the higher number we get in these two tables. 

**Table 1:** Quantitative evaluation of segmentation results for the five methods

**Method**	**SN**	**SP**	**PPV**
_DRLSE_	0.97±0.02	0.88±0.02	0.78±0.08
_DRLSE with NLTV_	0.99±0.01	0.94±0.02	0.89±0.04
Bression’s method	0.81±0.10	0.91±0.06	0.80±0.07
Bression’s method with NLTV	0.87±0.04	0.93±0.05	0.87±0.07
Our method	0.96±0.02	0.95±0.02	0.91±0.04

The average and standard deviation of the SN, SP and PPV across the kidney US images are provided.

**Table 2:** Quantitative evaluation of segmentation results for four methods

**Method**	**SN**	**SP**	**PPV**
_DRLSE_	0.95	0.87	0.81
_DRLSE with NLTV_	0.94	0.93	0.88
Our method without AM	0.89	0.87	0.79
Our method with AM	0.92	0.96	0.95

The value of the SN, SP and PPV are provided.

Compared with the original DRLSE method, our method makes an increasement by 1%, 7% and 13% on the SN, SP and PPV value respectively. And compared with the original level set with shape priors, out method makes an increasement by 15%, 1% and 14% on the SN, SP and PPV value respectively. The experiment results show our framework is the best.

With the help of the NLTV denoising method, the original DRLSE method get an increasement by 2%, 6% and 11% on the SN, SP and PPV value respectively, the original level set with the shape prior get an increasement by 6%, 2% and 7% on the SN, SP and PPV value respectively. From experiment results, we can see that the NLTV denoising method is important for the segmentation of ultrasound images.

When using the same initial process of the NLTV denoising method, out method still obtains an increasement by 9%, 1% and 4% on the SN, SP and PPV value respectively. This shows that our way of using shape prior is better than the common method.

Although we can get a promising result by integrating the shape prior into level set,our segmentation method sometimes does not get a satisfied result due to the lack of enough shapes in the shape space. While we use the alignment model to generate all kinds of shape, we can get an increasement by 3%, 9% and 16% on the SN, SP and PPV value.

## Conclusions

In this paper, we have presented a novel framework in the kidney segmentation of US images, dedicated to kidney surgery. NLTV denosing method, DRLSE and shape prior were combined in our framework. In order to validate our framework, we have quantitatively and qualitatively compared our method with other methods in literature. Disregarded the fact of having three steps in the framework, every step celebrated light computational burden and avoided the negative impact such as speckle noise and long computational time caused by the complicated iteration in the methods such as Bression’s. Furthermore, each step has shown to be able to improve the segmentation results effectively. Many methods have tried to reduce the noise influence by introduce more priors. However, from our experiments, we can see that simple level set method with preprocessing of NLTV denosing gets a good result. In addition, better results with smooth boundary can be available using the shape prior. Moreover, with the application of alignment model, our shape space has been effectively enlarged for better result. In our experiments, we can see that it’s better to use the shape prior in the end of the level set evolution to avoid the local minimum caused by the complicated.

## Methods

**Figure 4:**
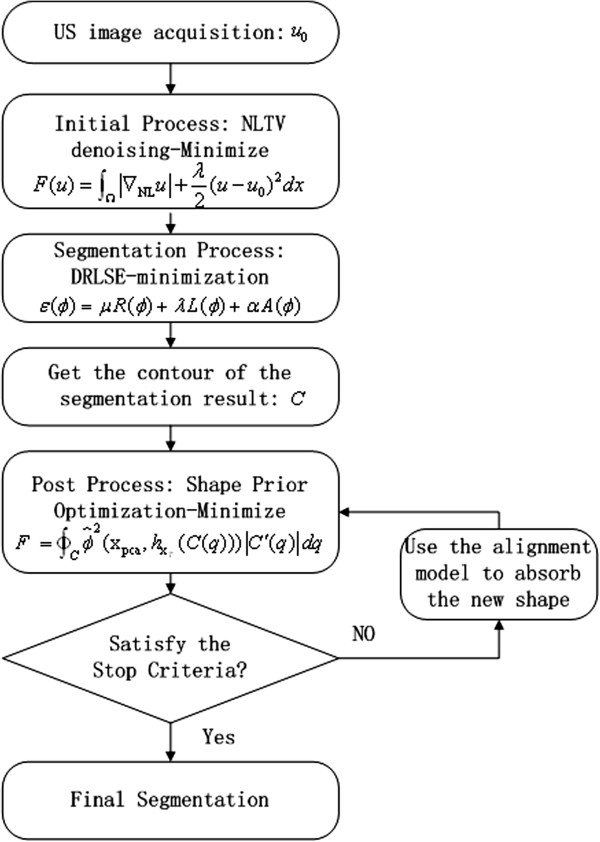
The proposed kidney segmentation framework.

The framework of the proposed approach is illustrated in Figure 4. The segmentation is three-fold: a specific preprocessing of US images to obtain denosing images, coarse segmentation of the kidney. an efficient optimization process of segmentation result with smooth boundary have been implemented. Sometimes the shape model may not cover all the shapes to get the satisfied result, so we can use the alignment model to generate a new shape to cover all kinds of deformed shape. 

### Initial process: NLTV denoising

US images are known to be low quality due to speckle or acounstic shadow. The aim of preprocessing US images is to reduce the noise, while preserving the echogenic boundaries of the organ. In this paper, we use NLTV denoising method to get a denoised image. Zhou-Scholkopf [19] and Gilboa-Osher [20] have introduced the definitions about nonlocal/graph (NL/G) gradient, which are useful in explaining the NLTV denosing model. The NL/G gradient of a function u:Ω→R is defined for the pair of points (*x*, *y*) ∈ *Ω* × *Ω* as:

(1) $$\nabla_{\text{NL}}u\left(x,y\right)=\left(u\left(y\right)-u\left(x\right)\right)\sqrt{w\left(x,y\right)}:\Omega \times \Omega \to R$$

Where u:Ω→R is the given image, such as a noise image, $w:\Omega \times \Omega \to R_{+}$ is the edge weight between the points *x*, *y* (*w* defines a graph), function *w* is defined as:

$$w\left(x.y\right)=e^{-\int_{\Omega }f\left(x,y,z\right)dz/h^{2}}$$

Where *f*(*x*, *y*, *z*) = ∫ _*Ω*_*G*_*a*_(*z*)|*u*_0_(*x* + *z*) − *u*_0_(*y* + *z*)|^2^*dz* is the distance between the points *x* and *y*, *G*_*a*_ is a Gaussian function with standard deviation *a*, and *h* is a Positive constant which acts as a scale parameter. The NL/G gradient is to give a weighted value between two points *x*, *y* to indicate whether the patches around point *x* and patches around point *y* are the same.

We utilized the nonlocal image denoising model of Gilboa-Osher [20]. Gilboa-Osher has used the NLTV operator in the form of ∫ _*Ω*_| ∇ _NL_*u*|. The whole image denoising model is written as follows:

(2) $$\begin{array}{l} \underset{u}{\inf}F(u)=\int_{\Omega }\left|\nabla_{\text{NL}}u\right|+\frac{\lambda }{2}{\left(u-u_{0}\right)}^{2}dx\\ \;=\int_{\Omega }\sqrt{\int_{\Omega }{\left(u\left(y\right)-u\left(x\right)\right)}^{2}w\left(x,y\right)dy}+\frac{\lambda }{2}{\left(u-u_{0}\right)}^{2}dx\; \end{array}$$

where *u*, *u*_0_: Ω→R, ∇ _NL_*u*: Ω×Ω→R, and *u*_0_ is the given noisy image and *λ* is a positive constant that controls the trade-off Between the regularization process and the fidelity with respect to the original image.

In order to get the denoised image *u*_*opt*_, we have to minimize Equation (2). Here, Zhang-Burger-Bression- Osher’s model [21] was used to solve the problem of NLTV minimization. More details can be referred to [21]. This initial process helped us to acquire an image with the kidney region in almost homogenous intensity gray scale. This is very beneficial for the following segmentation.

### Segmentation process: DRLSE

In level set method, the contour concerned is embedded as the zero level set of a level set function (LSF). During the evolution of level set, LSF may not be smooth. It may become steep or flat which destroy the unique property | ∇ *ϕ*| = 1. As a result reinitialization is in need. The most popular method is proposed in [22]. In this section, we used a level set formulation that has an intrinsic mechanism of maintaining this desirable property of the LSF. This formulation is called DRLSE proposed by Chunming Li [6].

Let *I* be an image on a domain *Ω*, edge indicator function *g* is defined by

(3) $$g≜\frac{1}{1+{\left|\nabla G_{\sigma }*I\right|}^{2}}\;$$

Where *G*_*σ*_ is a Gaussian kernel with a standard deviation *σ*. The convolution in (3) is used to smooth the image to reduce the surplus noise. This function *g* usually takes smaller values at object boundaries than at other locations.

For an LSF ϕ:Ω→R, energy functional *ε*(*ϕ*) is defined by

(4) $$\varepsilon \left(\phi \right)=\mu R\left(\phi \right)+\lambda L\left(\phi \right)+\alpha A\left(\phi \right)\;$$

Where *μ* > 0 *λ*>0 and α∈R are the coefficients of the energy functionals *R*(*ϕ*), *L*(*ϕ*) and *A*(*ϕ*) which are defined by

(5) $$R\left(\phi \right)≜\frac{1}{2}\int_{\Omega }{\left(\left|\nabla \phi \right|-1\right)}^{2}dx\;$$

(6) $$L\left(\phi \right)≜\int_{\Omega }g\delta \left(\phi \right)\left|\nabla \phi \right|dx\;$$

And

(7) $$A\left(\phi \right)≜\int_{\Omega }gH\left(-\phi \right)dx\;$$

Where *δ* and *H* are the Dirac delta function and the Heaviside function, respectively. This energy functional (4) can be minimized by solving the Following gradient flow:

(8) $$\frac{\partial \phi }{\partial t}=\mu \mathrm{div}\left(\left(1-\frac{1}{\left|\nabla \phi \right|}\right)\nabla \phi \right)+\lambda \delta \left(\phi \right)\mathrm{div}\left(g\frac{\nabla \phi }{\left|\nabla \phi \right|}\right)+\alpha g\delta \left(\phi \right)\;$$

Given an initial LSF *ϕ*(*x*, 0) = *ϕ*_0_(*x*), the first term on the right side in (8) is associated with the distance regularization energy *R*(*ϕ*) (5) which keeps the unique property LSF | ∇ *ϕ*| = 1. The second is associated with the energy terms *L*(*ϕ*) (6) which is minimized when the zero contour of *ϕ* is located at the object boundaries, while the third term is related to *A*(*ϕ*) (7) which is introduced to speed up the motion of the zero level contour in the level set evolution process. The segmentation process produced a binary image with the black and white region representing the kidney and background respectively.

### Post process: shape prior optimization

To model the shape prior, we applied a similar shape model construction method described in [4], [16] and [17]. This shape model is based on the principal component analysis (PCA). So we can obtain the main variations of a training set in which alignment model is applied,while removing redundant information. The new idea introduced in [4] is to apply the PCA on the signed distance functions (SDF) of these contours which are implicit and parameter free representations, instead of the parametric geometric contours or active shape model. This PCA method is able to produce a new kidney shape based on the training set {*ϕ*_*j*_}:

$$\hat{\phi }=\bar{\phi }+{\text{W}}_{p}{\text{x}}_{\text{pca}}$$

where x_pca_ is called the coefficients of eigen vector, $\hat{\phi }$ is a shape formed from the shape space, $\bar{\phi }$ is the mean shape of the shape space, and matrix W_*p*_ contains *p* principal components of the shape space.

Shape prior is to find the interesting shape in the binary image produced by the DRLSE method. The shape energy model proposed in [8] is as follows:

$$F_{\text{shape}}={\oint_{C}\hat{\phi }}^{2}\left({\text{x}}_{\text{pca}},h_{{\text{x}}_{T}}\left(C\left(q\right)\right)\right)\left|C^{\prime }\left(q\right)\right|dq$$

Functional *F*_shape_ is based on the fixed contour *C*, the vector x_pca_ of PCA eigen coefficients and the vector x_*T*_ of geometric transformations. This functional evaluates the shape difference between the contour *C* after the geometric transformations *x*_*T*_ and the zero level set $\hat{C}$ of the shape function $\hat{\phi }$ provided by the PCA. The function ${\hat{\phi }}^{2}$ at the point *C*(*q*) is:

$$\begin{array}{c} {\hat{\phi }}^{2}({\text{x}}_{\text{pca}},h_{{\text{x}}_{T}}(C(q)))={\hat{\phi }}^{2}({\text{x}}_{\text{pca}},C(q))≃{\left|{\hat{C}}_{{\text{x}}_{\text{pca}}}\left(p_{\min}\right)-h_{{\text{x}}_{T}}C\left(q\right)\right|}^{2} \end{array}$$

where | · | stands for the Euclidean norm.

The flow minimizing *F*_shape_ with respect to the vector of eigen coefficients x_pca_ is :

(9) $$\left\{ \begin{array}{l} d_{t}{\text{x}}_{\text{pca}}(t)=-2\int_{0}^{1}\hat{\phi }\nabla_{{\text{x}}_{\text{pca}}}\hat{\phi }\left|\nabla \varphi \right|\delta \left(\varphi \right)d\Omega {\text{x}}_{\text{pca}}(t=0)={\text{x}}_{T_{0}}\;\text{in}\;\Omega_{T} \end{array}\;\right.$$

and *φ* is a constant representing the signed distance function of the shape in the binary image.

The flow minimizing *F*_shape_ with respect to the vector of geometric transformation x_*T*_ is:

(10) $$\left\{ \begin{array}{l} d_{t}{\text{x}}_{T}=-2\int_{\Omega }〈\nabla \hat{\phi },\nabla_{{\text{x}}_{T}}h_{{\text{x}}_{T}}〉\hat{\phi }\left|\nabla \varphi \right|\delta \left(\varphi \right)d\Omega \;{\text{x}}_{T}(t=0)={\text{x}}_{T_{0}}\;\text{in}\;\Omega_{T} \end{array}\right.$$

For one iteration, the computation order is in the following order: (10), (9).

### Compare with other methods

**Figure 5:**
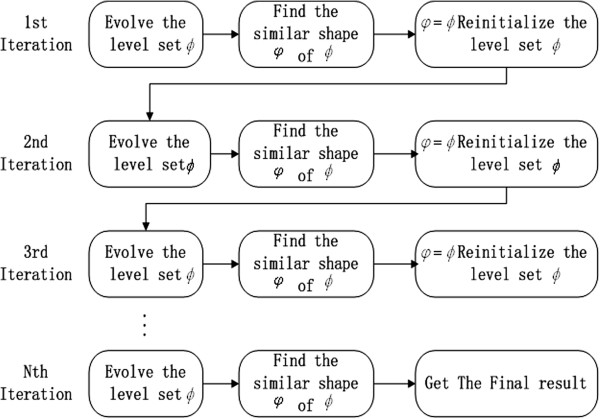
The flow chart of my method.

The segmentation process of [1,3,4,16-18] is shown in the Figure 5. The iteration procedure of these methods as follows: Firstly, they evolve contour level set according to the image texture, image gradient or region. Secondly, a similar shape of the evolving contour is found. Finally, reinitializing the level set is needed to keep its unique property | ∇ *ϕ*| = 1. In the every iteration, they find the interesting shape. 

**Figure 6:**
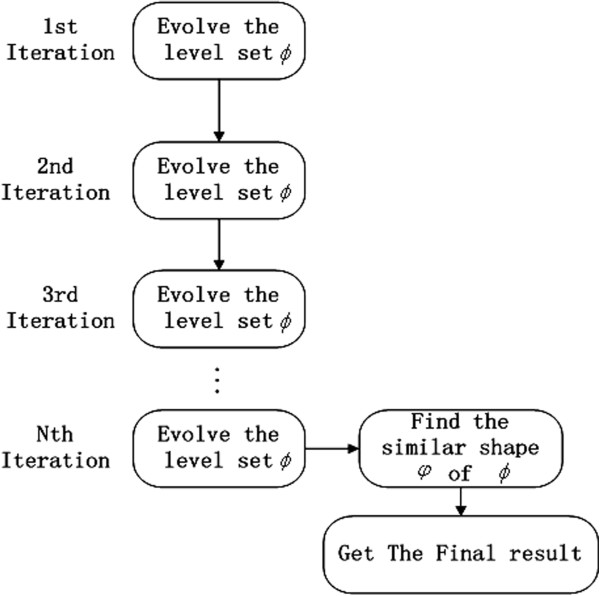
The common flow chart of level set with shape prior.

As the segmentation flow chart of our method is depicted in the Figure 6, we can observe that we only evolve contour level set in the every iteration. We do not need reinitialize the contour, because DRLSE has an intrinsic mechanism of maintaining this property of the LSF | ∇ *ϕ*| = 1. When the contour evolution is terminated, we find the interesting shape from the shape. Compared with the other method in [1,3,4,16-18], we do not need to search the interesting shape in every iteration and reinitialize the level set function. Thus, we save a lot of computation time. 

Compared with the original level set or DRLSE methods, we use the shape prior to optimize the segmentation result. Because the ultrasound image is low quality with speckle noise, the original level set or DRLSE method usually gets the segmentation result with indent boundary. In our framework, we use the shape prior to get a satisfied segmentation result with smooth boundary.

Due to the diversity in patients’ kidney shapes, the training sets used in the construction of the kidney shape space may not be enough. In many level sets with shape prior method [4,16-18], they had only used the similar model like the alignment model in the process of shape space construction. Once the shape space was established, it remained unchanged. However, in many situations, we cannot cover all kinds of shape from the initial data set. Hence the alignment model should be used to generate a new shape in order to give more kind of the shape space after the segmentation. In our process, when the SN, SP and PPV of the segmentation results all fell under 90%, we used the alignment model (AM) proposed in [18] to reconstruct the shape space and the new procduced shape was done by manual segmentations.

## Competing interests

The authors declare they have no competing interests.

## Authors’ contributions

FY suggested the algorithm for images analysing and processing, implemented it and analysed the images. QWJ and WTX performed the acquisition of the ultrasound images of kidney images and manuscript modified. GJ and XYQ expressed opinions on the evaluation metric of the segmentation results. All authors have read and approved the final manuscript.
